# Systematic, balancing gradients in neuron density and number across the primate isocortex

**DOI:** 10.3389/fnana.2012.00028

**Published:** 2012-07-18

**Authors:** Diarmuid J. Cahalane, Christine J. Charvet, Barbara L. Finlay

**Affiliations:** ^1^Center for Applied Mathematics, Cornell UniversityIthaca, NY, USA; ^2^Department of Psychology, Cornell UniversityIthaca, NY, USA

**Keywords:** cortex, cortical areas, cytoarchitecture, evolutionary development, gradient, neurogenesis, primate evolution

## Abstract

The cellular and areal organization of the cerebral cortex impacts how it processes and integrates information. How that organization emerges and how best to characterize it has been debated for over a century. Here we demonstrate and describe in the isocortices of seven primate species a pronounced anterior-to-posterior gradient in the density of neurons and in the number of neurons under a unit area of the cortical surface. Our findings assert that the cellular architecture of the primate isocortex is neither arranged uniformly nor into discrete patches with an arbitrary spatial arrangement. Rather, it exhibits striking systematic variation. We conjecture that these gradients, which establish the basic landscape that richer areal and cellular structure is built upon, result from developmental patterns of cortical neurogenesis which are conserved across species. Moreover, we propose a functional consequence: that the gradient in neurons per unit of cortical area fosters the integration and dimensional reduction of information along its ascent through sensory areas and toward frontal cortex.

## Introduction

Two hypotheses about the fundamental organization of the cerebral cortex (or, isocortex or neocortex) define the poles of a debate which has endured for close to a century. Crisp borders drawn on the cortical surface to delimit areas with distinct cellular architecture, as first penned by Brodmann and von Economo, seeded the “modularist” paradigm (Brodmann, 1913; Economo and Koskinas, 2008). In this view, each cortical region is described by its unique input and output connectivity, along with intrinsic circuitry corresponding to a particular type of data integration and transformation, the output of which is recombined in subsequent areas (Kaas et al., 2002). Even if sharply defined borders are no longer expected between most areas (Rosa and Tweedale, 2005), current functional imaging studies suggest that specific cortical regions can be associated with distinct cognitive tasks, isolating areas for identifying faces (Kanwisher et al., 1997), for judging others' actions (Saxe and Kanwisher, 2003), for linguistic functions (Fedorenko et al., 2011) and so on. Gene expression studies have sought to discover molecular mechanisms which imprint these areas on the developing cortex (Fukuchi-Shimogori and Grove, 2001; Sansom and Livesey, 2009; Yamamori, 2011) in the spirit of the “protomap” hypothesis (Rakic, 1988).

An alternative paradigm, initially “connectionist” (Elman et al., 1998) but which has extended to various second-generation architectures and network analyses, has roots in the mass action hypothesis of Lashley (1931) and echoes the “protocortex” model (O'Leary, 1989). It discounts the role of genetically determined regions and it highlights the uniform nature of the transformation and recombination performed by the cortex on any input. Neuroimaging studies in support of this view note the distributed and often redundant nature of cortical activation during most operations (O'Toole et al., 2005; Smith et al., 2009; Anderson, 2010). The connectionist paradigm envisions the embryonic cortex as largely undifferentiated; as synaptic connections form and refine under the influence of sensory input and endogenous cortical activity, areal specializations emerge (Johnson and Vecera, 1996).

A third paradigm is now emerging in which systematic variation across the cortical sheet may force the roughly hierarchical integration of information as is seen to occur both within and across sensory and motor systems (Felleman and Van Essen, 1991; Barone et al., 2000). We will argue that such variation derives from a conserved pattern of neurogenesis. The protomap and protocortex are increasingly recognized as not mutually exclusive and as both being important concepts for understanding the emergence of order in the cortex (Dehay and Kennedy, 2007). The paradigm we present here promises to contribute another key concept to our understanding of cortical development and organization.

**Figure 1:**
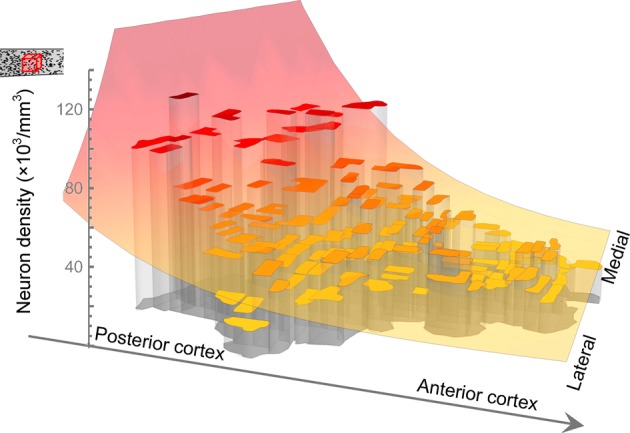
**Fitting a model to neuron density recorded in each sample in baboon.** Collins et al. removed and flattened the entire cortical sheet, cut it into samples and measured the density of neurons and number of neurons in each. The outlines of the samples of Collins et al. are as represented here and the height of each surface indicates the density of neurons measured in the corresponding sample. Also illustrated is the model function, increasing along an axis from anterior-lateral to posterior-medial cortex, which we have fitted to describe the global trend in neuron density.

The density and number of both neurons and glial cells across the isocortical expanse were analyzed with respect to known cortical areas in a recent study using the isotropic fractionator method (Herculano-Houzel and Lent, 2005) in four primate species (the galago, *Otolemur garnetti*; the owl monkey, *Aotus nancymae;* the macaque, *Macaca mulatta*; and the baboon, *Papio cynocephalus anubis*) (Collins et al., 2010a; Collins, 2011). The investigators noted that primary sensory areas in the isocortex had higher neuron numbers than other regions and noted other inter-areal variability along with potential phylogenetic and niche-related variability. However, they did not comment on what appeared to be pronounced anterior-to-posterior (or, more precisely, anterior-lateral to posterior-medial) gradients both in neuron density and in the number of neurons under a unit area of cortical surface (see Figure 1). For brevity, we will refer to the latter quantity as “neurons per unit column,” but we wish to be clear that our definition is independent of anatomical or functional definitions of a “cortical column.” In this report we analyze the data published by Collins et al. to demonstrate and describe those gradients in neuron number and density. Furthermore, we add data collected by microscopy in sectioned material from four additional New World monkey species. Not only do we find analogous gradients in those species, but the morphological detail and precise location of neurons that we describe in sectioned material also complements the data obtained using the isotropic fractionator method.

## Results

**Figure 2:**
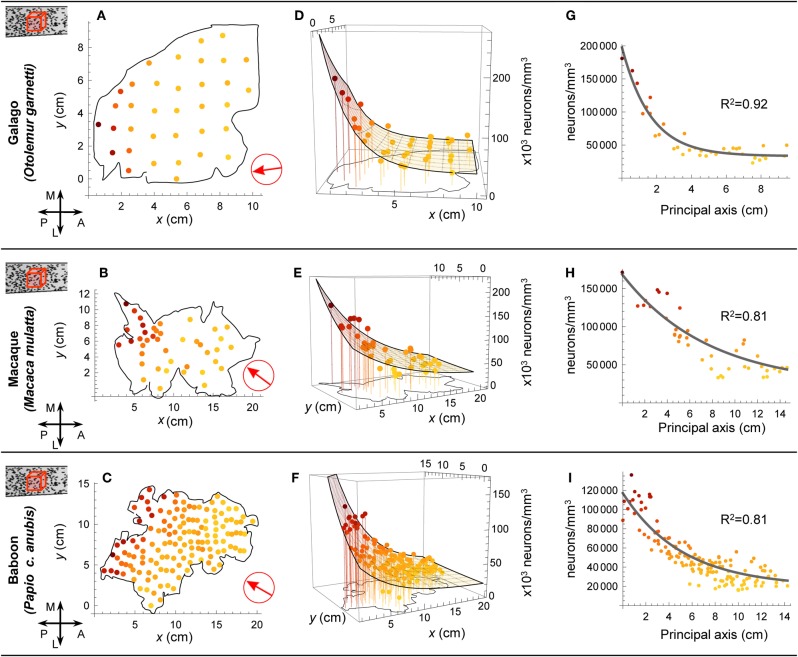
**Modeling neuron density.** In three primate species Collins et al. dissected the cortex into multiple samples and recorded the density of neurons in each dissected piece. In **(A**, **B,** and **C)** we denote the locations of the samples on the flattened cortical sheet. We fitted model surfaces **(D,E,F)** which allowed us to project the data onto a principal axis of variation **(G,H,I)**. In **(A,B,** and **C)** the red arrow indicates the alignment of the principal axis. A: anterior; P: posterior; M: medial; L: lateral.

**Figure 3:**
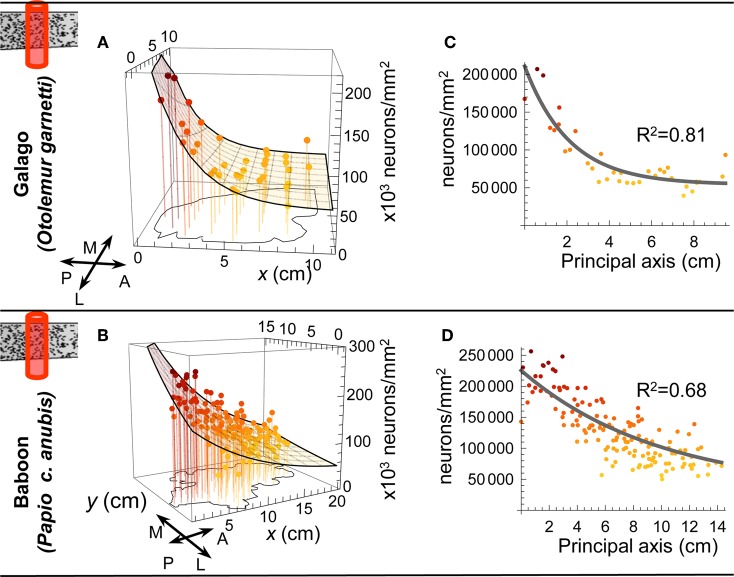
**Neurons per unit column. (A** and **B)** Model surfaces fitted to the to the number of neurons under a square millimeter of cortical surface in each of the samples tested by Collins et al. **(C** and **D)** As for the neuron density measurements (see text), we found that the data were well represented by projecting onto a single “principal axis.” A: anterior; P: posterior; M: medial; L: lateral.

The data collected by Collins et al. (Collins et al., 2010a) are plotted in Figure 2 (neuron density in galago, macaque, and baboon) and Figure 3 (number of neurons per unit column in galago and baboon). To collect these data for the galago and baboon the isocortical sheet was removed, flattened and cut orthogonal to the cortical surface into multiple samples having an approximately equal (top) surface area. The macaque cortex was processed similarly, but in this case the samples were cut along the borders of cortical areas, identified by reference to a map of known areas, before the cortex was flattened. Samples were processed using the isotropic fractionator to determine the number of neurons in each. The method is discussed in detail elsewhere (Collins et al., 2010b) but, briefly, entails homogenizing the samples, creating an isotropic suspension of dissociated nuclei, staining the dissociated nuclei and then counting them either under a microscope or with a flow cytometer. For each sample, its weight, density of neurons, total number of neurons and location in the cortical sheet were reported. For galago and baboon only, the (top) surface area of each sample was also reported.

To characterize the reported variation in the density of neurons and in the number of neurons per unit column across the cortical sheet, we fitted model surfaces (see Materials and Methods). Much of the variation in each dataset occurs as a super-linear trend along a single direction, which we will term the “principal axis” (see Figures 2 and 3). To model that variation, we chose functions from a family whose members' level sets are straight lines orthogonal to that principal axis—loosely speaking, the resulting surfaces are ramps, rising along the direction of the principal axis with increasing slope (see Figure 1). We used the same functional form to model neuron density in the galago, macaque and baboon (Figures 2D,E, and F), and the number of neurons per unit column in the galago and baboon (Figures 3C and D). Typically, the principal axis was found to point in an anterior-lateral to posterior-medial direction on the cortical sheet.

**Figure 4:**
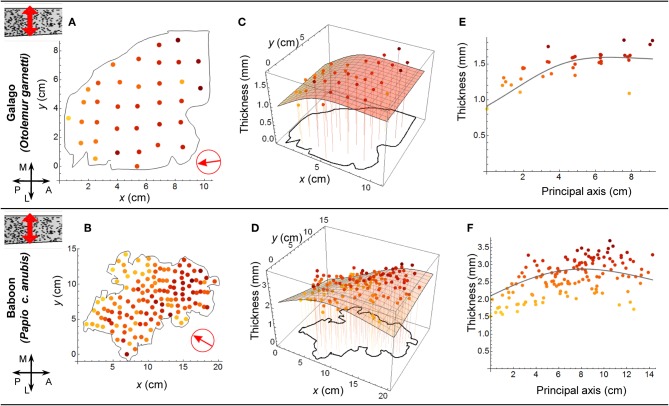
**Cortical Thickness.** Sample average thickness of cortex as calculated by dividing the number of neurons per column by the density of neurons for each sample in the study by Collins et al. Cortical thickness is seen to, on average, be reduced in posterior regions. The surfaces (in **C** and **D**) and the curves (in **E** and **F**) were calculated by dividing our respective model functions for neurons per column by those for neuron density. The arrows in (**A** and **B**) indicate the orientation of the principal axes.

**Figure 5:**
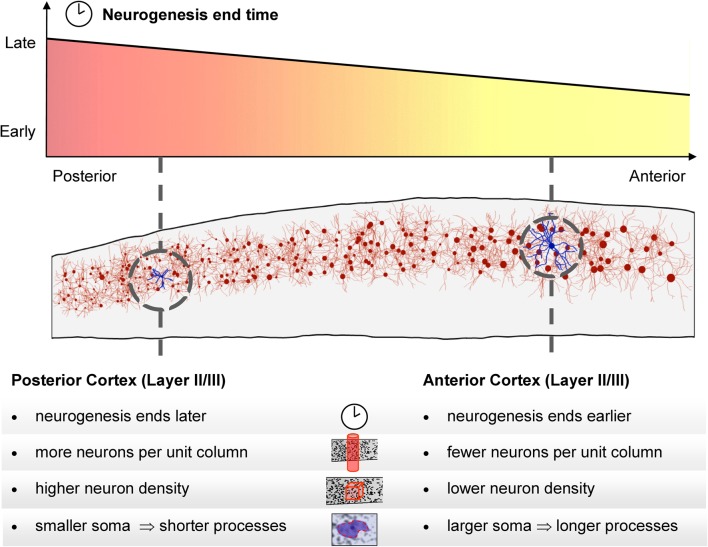
**Schematic depiction of the neurogenesis timing gradient and balanced gradients in neuron density and number per unit column.** In posterior regions neurogenesis continues for longer, resulting in a higher total number of neurons in each unit column. Higher neuronal density in those regions means that the increased number of neurons does not result in greater cortical thickness. We also found that the average size of a neuron's cell body in cortical layers II and III increases toward anterior regions. Larger neuron cell bodies are associated with longer axonal and/or dendritic processes.

For the galago and baboon, where both neuron density and number of neurons per unit column were available, the axes of variation of both those quantities were found to align to within a fraction of a degree (see Materials and Methods). Assuming a constant specific gravity of cortical tissue (Stephan et al., 1981), the collinear trends in the density and number of neurons in a unit column have consequences for cortical thickness along that same axis (Figure 4). Posterior cortex, despite having more neurons per unit column, is nevertheless thinner, on average, as a result of increased neuron density (see Figure 5 for a schematic summary). Thus, our analysis of the isotropic fractionator data reveals unambiguous global trends capturing a large fraction of the variance in the neuron density and number of neurons per unit column in the isocortex. Consistent with MRI (Fischl, 2000) and stereological (Pakkenberg and Gundersen, 1997) studies of human cortical thickness, we find that average cortical thickness varies over a much lesser range (by approximately two-fold) than do the underlying density and number of neurons (approximately five-fold)—a fact which may explain why such striking gradients spanning the cortex have gone unnoted.

**Figure 6:**
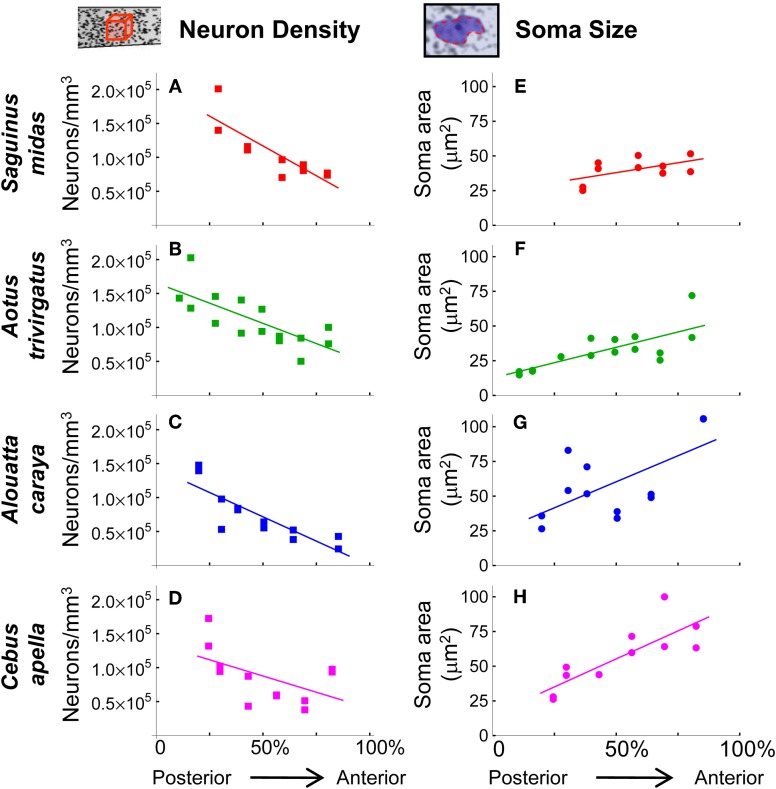
**(A–D)** Stereological measurements of neuron density in four species of New World monkeys. Neuron density decreases toward the anterior. Linear regression confirms the high significance (*p* < 0.002, one-tailed *t*-test) of the trend in **(A,B,** and **C)**. For **(D)**, *Cebus apella*, (*p* = 0.08). **(E–F)** Neuron soma size in cortical layers II and III. Soma size increases toward the anterior isocortex (in **E**, *p* = 0.09; **F**, *p* = 0.0008; **G**, *p* = 0.03, **H**, *p* = 0.001 using a one-tailed *t*-test).

Are such gradients in neuron density and neurons per unit column common to the cortices of all primates or perhaps to a still larger phylogenetic group? To assess the generality of these features, we estimated neuron density in the isocortices of four New World monkeys (a golden-handed tamarin, *Saguinus midas*; a northern owl monkey, *Aotus trivirgatus*; a black howler monkey, *Alouatta caraya*; and a tufted capuchin, *Cebus apella)*. Using light microscopy we examined serial sections along the anterior-posterior axis (see Materials and Methods). In all four species, neuron density increased in progressively more caudal regions (Figures 6A–D). Taken together, the results from seven primate species suggest that these systematic cortical variations in neuron density are general to the primate order.

We sought to better understand what variations in neural architecture underlie the observed differences in neuron density. The differences in density across cortical locations could be due to one or both of two-factors. Firstly, the dosage of densely packed granule cells, particularly in isocortical layer IV, is known to vary across the cortex. Secondly, a varying amount of connectivity of pyramidal neurons, with their axonal and dendritic processes occupying relatively more space as connectivity increases, could contribute to reduced neuron density. Here we present evidence of such increased connectivity. Since isocortical layers II and III together are both an important source and target of intracortical axonal projections, and because the volume and extent of a neuron's processes can be a factor in determining the volume of its soma [e.g., Elston et al. (2009)], we measured the soma sizes of neurons found in layers II and III. In the four New World monkeys we examined sites distributed along the posterior-to-anterior axis selected in the same manner as those for the stereological measurements of neuron density reported above (see Materials and Methods). Figures 6E–H show that neuronal soma size in isocortical layers II and III increases as neuron density decreases from the posterior to anterior in these cortices. In three of the four species examined, the global trend in soma size reaches significance (*p* < 0.05, one sided *t*-test), but outliers are present in all cases. The noisy nature of the trend hints that local effects are also influencing soma size, e.g., axon length, cortical area and the effects of experience (pruning or enlarging arbors) are all known to affect soma size. However, the global trends are consistent with the hypothesis that decreased neuron density in anterior regions is due, at least in part, to the greater amount of neuropil produced by increased intracortical connectivity.

**Figure 7:**
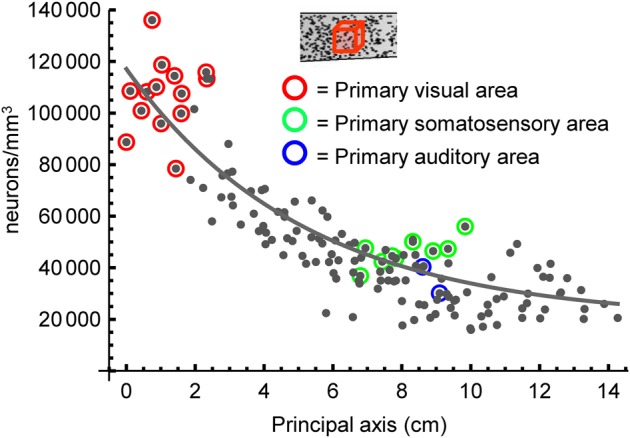
**Highlighting primary sensory areas in baboon.** Fitting all data points with a one-factor model (as described in the text) yielded the black curve. A two-factor model (not illustrated, see text) suggests primary sensory areas (those highlighted here) have an expected density 1.26 times greater than would a non-primary area in the same location.

To characterize the cortical architecture as varying systematically seems at odds with the notion that areas assume their properties idiosyncratically, prompted by genetic markers, projections from subcortical structures (Finlay and Pallas, 1989) or other locally present cues. For example, Collins et al. noted that areas involved in sensory processing had higher neuron densities than some adjacent areas (Collins et al., 2010a). Identifying the data points which related to primary sensory areas in the baboon, we noted that neuron densities at such sites typically lay above the model surface we had fitted (Figure 7). We used a two-factor model, incorporating each sample's location and whether or not it was from a primary sensory area, to show that primary areas have a density of neurons which is 1.26 times higher than that predicted for a non-primary area in the same cortical location. An *F*-test confirms that the two-factor model is the better descriptor of the data; [*F* = 28.3, *p* < 10^−6^, *d*.*f*. = (1, 135), see Materials and Methods]. Lower levels of neuron death during early development have been reported in putative sensory areas (Finlay and Slattery, 1983) relative to other areas and we suggest that this may contribute to the greater number of neurons per unit column in these regions. We offer this as an example of how local deviations are overlaid on the basic landscape set up by the global gradient in neuron number. We posit that the gradient itself is established by an isocortex-wide developmental pattern and acts in combination with more local mechanisms to develop the cortex's richer structure.

## Discussion

The global gradients in neuron density and number per unit column that we have described are matched with a prominent gradient of cortical neurogenesis that is conserved across species (see Figure 5 for a schematic summary). In every mammalian isocortex that has been studied, the non-cingulate isocortex is populated with neurons in an anterior-to-posterior progression, the progression being more pronounced in larger brains (Luskin and Shatz, 1985; Jackson et al., 1989; Bayer and Altman, 1991; Miyama et al., 1997; Kornack and Rakic, 1998; Rakic, 2002; Smart et al., 2002). For primates particularly, despite beginning at approximately the same time in all regions, neurogenesis generally ends earlier in frontal cortical regions than in posterior cortex (by as much as 3 weeks for some frontal regions in macaque, but with exceptions such as area 46) (Rakic, 1974, 2002). A neurogenetic interval of progressively longer duration in progressively more posterior regions, allowing a greater number of cell division cycles, would account for the greater number of neurons per unit column in those regions (Kornack and Rakic, 1998). Previously, it was suggested that in primates elevated levels of neurogenesis were specific to primary visual areas (Dehay and Kennedy, 2007). However, the location of the visual cortex, typically at the highest point on the density gradient, and the known reduction of cell death in primary sensory regions (Finlay and Slattery, 1983) may be sufficient to explain its high density of neurons. As to the lower density of neurons in anterior cortex, we offer the related developmental possibility that the earlier completion of neurogenesis in these regions may afford its neurons a head start and a lengthier interval to elaborate neuronal processes—it is known that isocortical neurons begin to establish their processes from the earliest stages of cortical development (Goldman-Rakic, 1987). That hypothesis is compatible with our finding of increased neuron soma size in cortical layers II and III, with studies showing enlarged pyramidal dendritic arbors in prefrontal cortex (Elston et al., 2009), and with the approximately constant number of synapses per unit volume across adult visual, auditory, and prefrontal cortex (Huttenlocher and Dabholkar, 1997). Process development should be distinguished from synaptogenesis however, as synapse formation appears to occur approximately simultaneously across the primate cortical sheet (Rakic et al., 1986).

Apart from aligning with a developmental axis, we point out that the cortical variations we have highlighted are also aligned with important functional and processing axes. The gradients have consequences for the makeup of neural circuitry, implying a rostral-to-caudal shift in the ratio of space apportioned to neurons' cell bodies versus their connective processes. So, it is of interest that higher stages of information processing and integration in the cortex occur at progressively more anterior locations. Higher visual areas and association areas integrating visual information are located anterior to the primary visual areas (Van Essen et al., 1992). The motor areas, which integrate somatosensory information in motor control, are located anterior to the primary areas receiving somatosensory input. However, such an alignment in the auditory processing areas is not so clearly evident (Kaas and Hackett, 2004). We note that the auditory areas differ from the other sensory areas in lacking a spatial topography and in occupying a much smaller proportion of the primate cortical surface. That small spatial extent means the global gradient in neuron number would imply little change in cellular architecture across these areas in any case. Network analysis of structural connectivity in the cortex also suggests an anterior-to-posterior gradient whereby frontal regions have more integrative roles, evidenced by the preponderance of network hubs being located in those regions (Modha and Singh, 2010). We conclude that the architectural gradients we have identified foster successively higher and more integrative stages of neural processing: as information is represented in successively higher (i.e., progressively anterior) areas, their reduced areal extents and lower numbers of neurons per unit column imply the dimensional reduction or other compression of that information. Considering the opposing direction, the gradients discussed here also align with established and hypothesized contra-flows of neural information issuing from frontal regions. It has been shown that progressively anterior regions of prefrontal cortex execute “progressively abstract, higher control” of behavior (Badre, 2008). The control of attention has been shown to propagate backward through visual areas (Buffalo et al., 2010). Merker has hypothesized that a “countercurrent” from frontal regions provides more caudal regions a context to associate with sensory information (Merker, 2004). The so-called Bayesian brain hypothesis also posits an anterior-to-posterior flow of context-relevant predictions about future input, priming relevant representations and ultimately acting on lower sensory areas to guide perception (Bar, 2007).

We wish to be clear that the cortex-wide gradient in cellular architecture we describe here does not preclude the presence of abrupt anatomical borders between cortical areas. Neither the data of Collins et al. nor the histology we report has sampled the cortical surface at sufficient density to resolve, for example, the border between visual areas 17 and 18, which is readily visible in stained sectioned material from primates even at low magnification. Our hypothesis is that cellular architecture changes in an ordered progression at the isocortex-wide scale. Examining the cortex with suitable methods at a higher resolution would refine local features such as areal borders. Perhaps a useful analogy is that of a staircase: to discuss to the overall slope or pitch of the staircase, a global measure, is not to deny the presence of discrete steps. Evidence for the modular, hierarchical genetic organization of the cortex at the intermediate scale of lobes and regions has been found by analysis of the cortical surface in twins (Chen et al., 2012).

The isotropic fractionator, being a high throughput method, is ideal for comparative studies examining large numbers of cortical loci. For example, the large number of samples examined by Collins et al. (*N* = 141 in baboon) and the uniformity of their distribution in the cortical sheet stand in contrast to the sampling of just six sites (V1, S1, M1, and Brodmann areas 7, 9, and 22) in an oft-cited report of the “basic uniformity” of cortical structure by Rockel et al. (1980). That report concluded the number of neurons per unit column is the same in all of non-visual cortex and constant across five species (mouse, rat, cat, macaque, and human). Rockel did find that the number of neurons in primary visual cortex in primates was elevated by a factor of 2.5 over that in other cortical areas, and the data of Collins et al. support a comparable contrast between primary visual areas and some neighboring areas, but there the similarities end. Under-sampling of the cortex by Rockel et al. may have contributed to their finding of constant neuron number, with just one of the six sites examined being in frontal cortex. Numerous previous studies have contradicted the conclusions of Rockel et al. regarding both within-cortex variation and cross species variation, e.g., Pakkenberg and Gundersen (1997); Beaulieu and Colonnier (1989); Cheung et al. (2007, 2010) and several others discussed in Collins et al. (2010a), but the definitive contribution of Collins et al. surely provides closure on this matter.

Despite the value of the isotropic fractionator as a comparative tool, it does have limitations. Firstly, it is unclear whether every neuronal nucleus present in the tissue samples survives the dissociation step, is successfully stained and then detected at the counting step. However, any under-counting of nuclei would result in the same fractional error in the estimated neuronal density of each sample. Thus, while the absolute number of neurons might be under-estimated, comparing estimates across samples is still useful. Secondly, the isotropic fractionator cannot tell apart different neuronal cell types, examine their morphology, nor identify the layers those neurons had occupied in the intact cortex. For that reason, traditional histology in sectioned material can usefully compliment results from fractionator studies by providing additional information at a subset of the cortical sites examined. For example, in sectioned material in the present study we identified those neurons in cortical layers II and III and estimated their soma size by tracing cell body outlines at high (60×) magnification. In future work, such methods will allow us to investigate in detail how each layer contributes to the gradient in neuron number and how neuron density varies within layers.

In summary, we emphasize the empirical finding that two gradients—an increase in the density of neurons and an increase in the number of neurons per unit column—align on an axis from the frontal to occipital poles of the mature primate cortex. The gradients are balanced in the sense that their net effect is to produce a cortex whose thickness changes, by comparison, to a much lesser extent. Variation in the cellular architecture across cortical regions surely also implies a corresponding variation in the types of neural processing tasks that regions are most apt to support. Understanding the interaction of the global variations we have described with local features, such as the presence of genetic markers or subcortical sensory projections, will be central to understanding how cortical areas assume and execute their respective roles in neural processing. To conclude, we propose that the modularist's vision of the embryonic isocortex as a patchwork and the connectionist's view of it as a blank computational canvas would be better replaced by the metaphor of a staircase, with position along the staircase having significance for the nature of the computation carried out there. The connectionist must acknowledge that not all steps are equal and the modularist must acknowledge their global trend. The entwined challenges of understanding the evolution, development, anatomy, function, and pathologies of the isocortex will surely demand such integrative perspectives.

## Materials and methods

### Species

This report includes an analysis of previously published data (Collins et al., 2010a; Collins, 2011) relating to a baboon (*Papio cynocephalus anubis*), a rhesus macaque (*Macaca mulatta*) and a prosimian galago (*Otolemur garnetti*) collected using the isotropic fractionator method. We collected original data in sectioned tissue from four species of New World monkeys: one golden-handed tamarin (*Saguinus midas*), one northern owl monkey (*Aotus trivirgatus*), one black howler monkey (*Alouatta caraya*), and one tufted capuchin (*Cebus apella*). These samples came from previous studies conducted in this laboratory (Kaskan et al., 2005; Chalfin et al., 2007). The animals had been bred or housed in the Centro Nacional de Primatas in Pará, Brazil. The sex, brain weight and specimen ID of these animals are listed Table 1.

**Table 1:** **Species data for New World monkeys specimens used in stereological measurement of neuron density and layer II and III soma size**.

**Species**	***Saguinus midas***	***Aotus trivirgatus***	***Alouatta caraya***	***Cebus apella***
Specimen ID	SM 970108B	AT 980115A	AC 970111A	CA 970913
Sex	F	F	M	M
Brain weight (g)	9.09	14	54.3	62
Hemisphere examined	Right	Left	Left	Right
Magnification	60×	60×/ 40×	40×	60×
Section thickness (μm)	60	60	60	60
Section thickness, shrunken (μm)	28	25	28	28.1
Number of sections examined	5	8	7	6
Guard zone (μm)	5	5	5	5

### Ethics statement

The original data in this report was collected from animals housed and treated in compliance with the principles defined in the National Institutes of Health Guide for the Care and Use of Laboratory Animals, as certified by Cornell University's Institutional Animal Care and Use Committee as part of a larger study.

### Sample preparation

For *Saguinus midas*, *Aotus trivirgatus, Alouatta caraya*, and *Cebus Apella*, the animals were adapted to dark conditions for 30 min while a light anesthetic was administered by intramuscular injection (a 1:4 mixture of 2% xylazine hydrochloride and 5% ketamine hydrochloride). The same preparation was then used to deeply anesthetize the animals. They were perfused with a phosphate-buffered saline solution (PBS) with a pH of 7.2 prior to perfusion with 4% paraformaldehyde. The brains were removed and weighed. One to two weeks later, the brains were stored in a 2% paraformaldehyde solution. Prior to sectioning, the brains were placed in a 30% sucrose/PBS 0.1 M preparation having a pH of 7.2. Coronal sections were made at 60 μm using a freezing microtome. Every fifth or seventh section was kept, mounted on a gelatinized slide and stained with cresyl violet.

### Estimating neuron density and neuron soma size

Sections were examined using a Leitz Diaplan micropscope and a Neurolucida imaging system with a mechanical stage (Mircrobrightfield Inc., Colchester, VT). Coronal sections were selected, approximately equally spaced along the rostral-caudal axis, excluding the most caudal and rostral sections (Figure 6). The number of sections chosen for each species is given in Table 1. We did not correct for shrinkage of the sectioned material—the within-cortex comparisons we present are unaffected by this. Cells with small and condensed somas were not included so as to exclude glial cells from the analysis.

#### Site selection

In each section, we randomly selected two regions in the right or left (see Table 1) isocortical hemisphere within which to estimate neuron size and density. Within the randomly selected regions, we overlaid a grid on the magnified image to randomly select a column of cortex. At the selected location, the axes of a grid were aligned to be (respectively) tangential and normal to the cortical surface at that location. For the purpose of this description, we refer to the axis normal to the surface at each sampling site as a “sampling axis.”

#### Neuron density estimates

**Figure 8:**
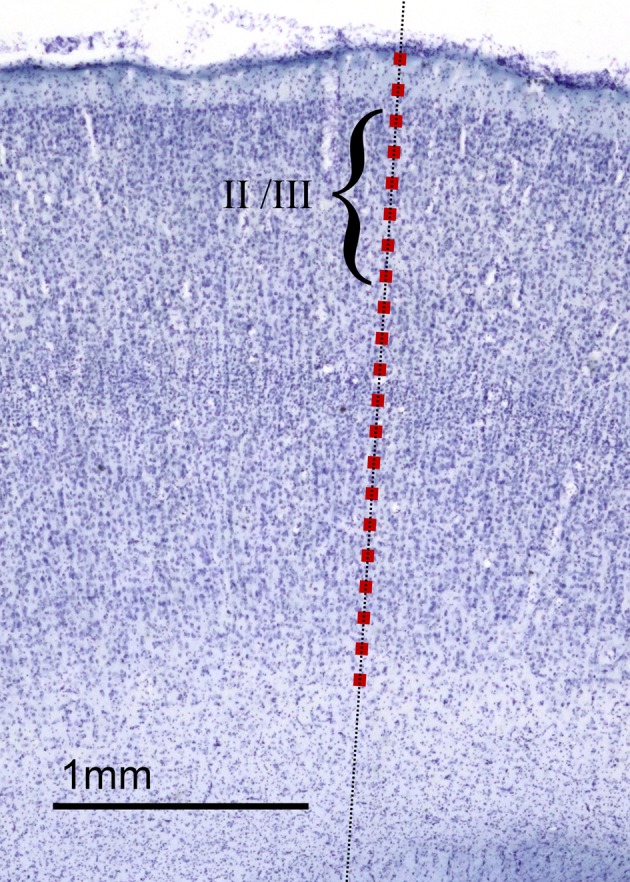
**Counting boxes for neuron density and neuron soma size estimates.** As outlined in the Materials and Methods section, and as illustrated here in a section from *Aotus trivirgatus* temporal isocortex, sampling axes (dashed line) were placed normal to the cortex's outer surface at chosen sites in cortical sections. Along each sampling axis, counting boxes measuring 41 μm × 41 μm (red squares, drawn to scale) were placed, typically at 100 μm intervals, from the surface to the white matter. Neuron density estimates were made within each counting box. In those counting boxes that lay in cortical layers II and III (indicated by the bracket), estimates of neuron soma size were also made.

We placed counting boxes measuring 41 μm × 41 μm at 50–200 μm intervals along each selected sampling axis, beginning at layer I and until the boundary between layer VI and the white matter was reached (see Figure 8). We used the optical disector method (Williams et al., 2003) to estimate the number of neurons contained in each box's volume. The 60 μm sections were thick enough to employ a three-dimensional, 5 μm thick guard zone, whereby neurons that lay on the three exclusion planes (*x*, *y*, and *z* planes) were not counted. Details of how many counting boxes were used to calculate density along each sampling axis are given in Table 2.

**Table 2:** **Numbers of sites along a sampling axis at which counts were made to determine neuron density**.

**Species**	***Saguinus midas***	***Aotus trivirgatus***	***Alouatta caraya***	***Cebus apella***
Mean number of locations sampled per column	17.4	16.1	18.8	20.1
Minimum number of locations sampled per column	6	8	10	8
Maximum number of locations sampled per column	27	27	32	45

#### Neuron soma size estimates

We estimated neuron size in isocortical layers II/III. Beginning at the layer I/layer II interface and ending at the layer III/layer IV interface, we placed counting boxes measuring 41 μm × 41 μm at intervals of 100 μm along the sampling axis. Within each box, once the focal plane was fixed we identified those neurons whose nuclei were clearly visible. This ensured only neurons were counted. Moreover, it ensured that our estimates of soma area were consistently made, using a cross-section of the neuron that contained the nucleus rather than an arbitrary cross-section. For the range and mean of the number of neurons selected per sampling axis see Table 3.

**Table 3:** **Numbers of layer II and III neurons measured along a sampling axis to estimate soma size**.

**Species**	***Saguinus midas***	***Aotus trivirgatus***	***Alouatta caraya***	***Cebus apella***
Mean number of neurons selected per column	13.8	15.1	9.8	14.6
Minimum number of neurons selected per column	6	9	6	8
Maximum number of neurons selected per column	19	25	13	26

### Mathematical and statistical methods

#### Modeling neuron density and number per unit column

In samples cut from the flattened cortical sheet, Collins et al. recorded neuron density and the total number of neurons along with the top surface area and a tracing of each sample's outline on the cortex prior to sectioning. We assigned Cartesian coordinates (*x, y*) to denote, in the two-dimensional plane of the flattened cortical sheet, approximately the centroid of each sample. For both the neuron density and neurons per unit column measurements, we noted in each species that (a) there was a super-linear trend in the data and (b) most of the variation in the data was in a roughly anterior-lateral to posterior-medial direction. For these reasons, we chose to fit the following surface (with fitting parameters *a, b, c*, and *d*) to quantify the trend and to identify the principal axis of variation: $f(x,y)=a+\exp[b+c(dx+(\sqrt{1-d^{2}})y)]$. This function increases as an exponential along one direction and is level along all lines parallel to the orthogonal direction. The direction of the principal axis of variation is given by the fitted parameter *d* via θ = arccos(*d*). In each case, we fitted the surface to the data by minimizing the sum of the squared errors using the “FindFit” function in Mathematica (Version 7, Wolfram Research, Champaign, IL.). The fitted values of the parameters, as well as the coefficient of determination *R*^2^ for each case, are as in Table 4. The results of projecting the data on to the principal axes are shown in Figure 2, parts g, h, and i, for neuron density and in Figure 3, parts c and d, for neurons per unit column. This provides visual confirmation that one axis captures much of the variance. To validate that observation, in each case we projected the model's residuals onto the orthogonal axis and carried out a linear regression to test for trends in the data. In no case was there a significant trend along the orthogonal axis (*p* > 0.15 and *R*^2^ < 0.07 in all cases).

**Table 4:** **Parameters for curve-fitting of neuron density and neurons per unit column**.

**Species**	**Measurement**	***N***	***a***	***b***	***c***	***d***	θ	*R*^2^
*Otolemur g*.	Neurons per column	35	5.41 × 10^4^	12.5	−0.476	0.989	8.6°	0.81
	Neuron density	35	3.33 × 10^4^	12.7	−0.613	0.989	8.4°	0.92
*Papio c. a*.	Neurons per column	141	3.16 × 10^4^	12.0	0.118	−0.858	−30.9°	0.68
	Neuron density	141	1.98 × 10^4^	11.1	0.225	−0.854	−31.3°	0.81
*Macaca m*.	Neuron density	41	2.21 × 10^4^	11.6	0.155	−0.839	−32.9°	0.81

N is the number of points in each data set. *R*^2^ is the coefficient of determination for each fit.

#### Two-factor model for neuron density

In the baboon neuron density dataset, we tagged each data point with a binary descriptor of whether or not it belongs to a primary sensory area (V1, S1, or A1). Collins et al. had identified the samples from such areas by viewing the flattened cortex on a light box, whereby myelin-dense sensory areas are opaque relative to the surrounding areas. Samples for which more than half of their surface area lay within a primary sensory area were tagged as belonging to that primary sensory area. We let *q*_*i*_ denote the density of neurons as measured in the *i*^th^ sample and let (*x*_*i*_, *y*_*i*_) be the sample's location. We let *s*_*i*_ equal 1 if the *i*^th^ sample belongs to a primary sensory area and let it equal 0 otherwise. We obtained our fit by adjusting the parameters *a, b, c, d*, and *e*, to minimize the quantity ∑_*i*_[(1 − *e* × *s*_*i*_)*q*_*i*_ − *f*(*x*_i_, *y*_i_)]^2^ with $f(x,y)=a+\exp[b+c(dx+(\sqrt{1-d^{2}})y)]$, as in the location-only model described above. This minimization amounts to carrying out a least squares fit of the location-only model with the added parameter *e* now discounting the densities of primary sensory areas. Loosely speaking, the discount term *e* quantifies by what fraction the density of primary sensory areas would need to be reduced to fall “in line” with their non-primary sensory neighbors. The result of fitting the two-factor model (using the “NMinimize” function in Mathematica) is shown in Table 5. The coefficient of determination, *R*^2^ = 0.84, is seen to be higher than in the location-only model (*R*^2^ = 0.81). We note that the location-only model is nested within the extended model (to see this, take *e* = 0), and so an *F*-test can be used to confirm the high significance of the improvement in the value of *R*^2^ [*F* = 28.3, *p* < 10^−6^, *d*.*f*. = (1, 135)]. The value of *e* = 0.206 yielded by the fitting procedure can be interpreted as primary sensory area having a density which is 1/(1 − *e*) ≈ 1.26 times greater than would be predicted in this model for a non-primary sensory area at the same location.

**Table 5:** **Parameters for fitting our two-factor model for baboon neuron density**.

**Species**	**Measurement**	***N*_*p*_**	***N*_*np*_**	***a***	***b***	***c***	***d***	***e***	θ	***R*^2^**
*Papio c. a*.	Neuron density	25	116	1.19 × 10^4^	11.0	0.158	−0.846	0.206	−32.2°	0.84

In this model we discount the recorded density at primary sensory areas by a factor of (1 − e). N_p_ is the number of data points from primary sensory areas and N_np_ is the number of data points from non-primary areas. R^2^ is the coefficient of determination.

### Conflict of interest statement

The authors declare that the research was conducted in the absence of any commercial or financial relationships that could be construed as a potential conflict of interest.
